# Psychometric Properties of the Chinese Version of the Brief Interpersonal Competence Questionnaire for Adolescents

**DOI:** 10.3390/children10010059

**Published:** 2022-12-27

**Authors:** Liuyue Huang, Junrun Huang, Zhichao Chen, Weiwei Jiang, Yi Zhu, Xinli Chi

**Affiliations:** 1 Department of Psychology, Faculty of Social Sciences, University of Macau, Macao 999078, China; 2 Center for Cognitive and Brain Sciences, Institute of Collaborative Innovation, University of Macau, Macao 999078, China; 3 School of Psychology, Shenzhen University, Shenzhen 518060, China; 4 School of Early-Childhood Education, Nanjing Xiaozhuang University, Nanjing 210017, China; 5 The Shenzhen Humanities & Social Sciences Key Research Bases of the Center for Mental Health, Shenzhen 518060, China

**Keywords:** social competence, measurement, adolescents, validity study

## Abstract

This study aimed to evaluate the psychometric properties of the Brief Interpersonal Competence Questionnaire (ICQ-15) administered to Chinese adolescents. A sample of 1705 adolescents (Mean age = 14.08, SD = 3.22, 46.5% male) completed a questionnaire including the Chinese version of the ICQ-15, as well as measurements of well-being, psychological resilience, and depression. To examine the psychometric properties of the ICQ-15, item analyses (item–total correlation and normality test), confirmatory factor analysis, concurrent validity analyses, multi-group analyses, and internal consistency analyses were performed. The results of the item analyses suggested a good item–total correlation, and the item scores were distributed approximately normally. The confirmatory factor analysis showed that the five-factor model had acceptable fit indices. The concurrent validity analyses indicated that the Chinese version of the ICQ-15 had a satisfactory concurrent validity. The multi-group analyses proved the measurement invariance across females and males, as well as participants in early, middle, and late adolescence. The ICQ-15 demonstrated satisfactory internal consistency reliability among Chinese adolescents. The ICQ-15 presents good psychometric properties and can be used to assess interpersonal competence in Chinese adolescents.

## 1. Introduction

Interpersonal relationships play an influential role in a person’s life-span development and well-being [1]. A growing body of research indicates that individuals with good interpersonal relationships have a higher sense of well-being, greater academic resilience, more adaptive coping styles, and healthier physical conditions [2,3,4]. In contrast, poor interpersonal competence may result in interpersonal conflicts and even lead to the progression of internalizing and externalizing behaviors [5]. Notably, adolescents are in a critical period of transition from childhood to adulthood, with the pursuit of independence from their parents and the need for more social interactions with their peers. As such, interpersonal competence becomes particularly important at this stage [6]. A meta-analysis of 51 studies in China found that 24.3% of Chinese adolescents suffer from depression symptoms [7]. After the outbreak of COVID-19, adolescents’ mental health problems have become a major concern in our society. A meta-analysis including 80,879 adolescents from around the world showed the pooled prevalence rate was 25.2% for depression and 20.5% for anxiety during the COVID-19 pandemic [8]. Local researchers are urged to pay special attention to adolescents’ mental health and explore ways to improve it.

A key approach to promoting adolescents’ mental well-being may be facilitating their interpersonal competence, whose lack has been shown to have a close negative association with mental health (e.g., depression) [9,10]. Similar to studies with adults, adolescents with deficient interpersonal competence were also found to be at a higher risk for loneliness, anxiety, and even suicidal behavior [11,12]. Conversely, adolescents with good interpersonal relationships are likely to experience fewer mental health problems and better school adaption and subjective well-being [13]. In this respect, interpersonal competence is particularly crucial for the healthy development of adolescents.

With the rise of research interest in adolescents’ interpersonal competence, well-validated measures have become indispensable. In addition to providing scientific tools for assessing adolescents’ interpersonal competence, scales with good reliability and validity can contribute to the evaluation of the effectiveness of related interventions. Indeed, interpersonal competence can be changed through interventions, particularly in the early years of life (i.e., childhood and adolescence) [14]. Existing studies have shown interventions targeted at social competence could improve adolescents’ interpersonal efficacy and mental health status [15,16]. However, interpersonal competence improvement is a long-term, dynamic process that requires continuous evaluation [17]. There are times when studies are conducted under constrained settings (e.g., assessment time and cost). For example, there are large-scale investigations on the comprehensive development status of students or intensive clinical progress follow-up studies. A refined scale is required to reduce the response burden of the participants in these research contexts. As a result, both research and practice require short-form scales that are validated to measure interpersonal competence.

Regarding the current assessments of individuals’ interpersonal relationships, there are two main categories. Most scales are disease-focused and based on the diagnosis systems of the International Classification of Diseases [18] or the Diagnostic and Statistical Manual of Mental Disorders [19]. The other category is from the perspective of positive aspects (e.g., strength and competence). To date, most existing scales for interpersonal relationships validated in the Chinese population were mainly designed to measure social problems, such as social anxiety and social phobia [20,21]. Notably, perspectives based on a “problem perspective” are prone to attach negative labels to the adolescents [22], which may have a negative influence on their self-identity.

In contrast, scales from the perspective of development are beneficial to reduce stigma. Several scales were developed to measure social competence from a positive perspective. For example, the 77-item Multidimensional Social Competence Scale showed acceptable psychometric properties among general young adults [23]. Nevertheless, too many questions may lead to burnout in large-scale testing and intensive evaluation. The Interpersonal Competence Questionnaire (ICQ), developed by Buhrmester and colleagues [24], is a widely used interpersonal competence measurement. The ICQ contains 40 items with five dimensions: initiation of a relationship, negative assertion, disclosure, emotional support, and conflict management. This questionnaire has been used in various countries and is now available in many different languages, including Chinese [25], German [26], Polish [27], Italian [28], and Korean [29], with good psychometric properties. However, the ICQ, with 40 items may be burdensome to administer in time- or cost-constrained conditions such as when performing comprehensive large-scale studies (e.g., adolescents development census) or intensive measurement studies (e.g., clinical progress follow-up) [17]. To increase the feasibility of the ICQ for routine assessments in research and clinical practice, Coroiu et al. [17] simplified and validated a 15-item version of the Interpersonal Competence Questionnaire (ICQ-15) on the basis of the ICQ. Consistent with the theoretical structure of the original scale, the ICQ-15 retained the five dimensions (three questions for each dimension). Not only the ICQ-15 showed good validity in the general adult population (age 18–90) but also facilitated the assessment of interpersonal competence under various environmental constraints.

The ICQ-15 has been validated in adolescent populations in Germany [17] and Spain [30], with the demonstration of good psychometric properties. However, the ICQ-15 has yet to be adapted to and validated in the Chinese adolescent population. As interpersonal competence is an essential part of the developmental process of adolescents, this study proposed to adapt the ICQ-15 and evaluate its reliability and validity among Chinese adolescents. To this end, the present study aimed to adapt the ICQ-15 to the Chinese context and to examine its validity in Chinese adolescents. We hypothesized that the Chinese version of IC1-15 has good psychometric properties.

## 2. Method

### 2.1. Participants

According to a recent proposition of the period of adolescence, adolescence spans the years between late childhood and early adulthood (ages 10 to 24 years, see [31]. The present study recruited participants aged 10 to 24 years. A total of 2100 questionnaires were distributed, and 1991 questionnaires were returned, with a response rate of 94.81%. The valid sample size was 1705 after excluding 291 invalid questionnaires, due, for example, to too short response times or invalid answers (e.g., all items were given the same answer or did not pass the attention check test). The control of the response time was performed after participants’ submission according to the answering time recorded by the system. The online survey platform has the function of recording the participants’ response time. According to the length of the survey, those participants who completed the whole questionnaire in less than 5 min were considered to be invalid cases. The mean age was 14.08 years (SD = 3.22). There were 793 (46.5%) male students and 912 (53.5%) female students. Regarding the school level, 271 (15.9%) were college students, 272 (16.0%) were high school students, 552 (32.3%) were middle school students, and 610 were elementary school students (35.8%).

### 2.2. Procedure

We used a convenience sampling method to collect data from May to June 2021 in five schools in Shenzhen, China, including one elementary school, one secondary school, two 12-year schools (primary to senior high school), and one university. Students from elementary, middle, and high schools were a cluster convenience sample. The research team collaborated with the school institutions and recruited most students in these sampling schools. The university participants were recruited by snowball sampling. The university student population was recruited by means of posters on university school web forums and social media platforms (e.g., WeChat, QQ). Moreover, no incentive was provided to the participants from primary to senior high school. Participants from the university were offered the opportunity to enter a draw for RMB 1~100, cash (equivalent to USD 0.14~14 at the time of the assessment). All participants could decide to withdraw from the study at any time. All participants included in the final analyses provided informed consent. All data were collected online via Sojump (https://www.wjx.cn/) (accessed on 8 December 2022), a widely used online questionnaire platform in China. There were no missing data in this study because all items needed to be completed before submission. This research project was approved by the Ethics Committee of Shenzhen University (No:2020005).

### 2.3. Instruments

#### 2.3.1. Brief Form of Interpersonal Competence Questionnaire (ICQ-15)

ICQ-15 [17] is the short version of the widely used ICQ [24], with 15 items containing 5 dimensions: initiation of a relationship (items 2, 7, 9), negative assertion (items 10, 12, 13), disclosure (items 3, 5, 8), emotional support (items 4, 11, 14), and conflict management (items 1, 6, 15). The participants were invited to rate these items according to how challenging each social behavior was for them. The answers were scored on a 5-point Likert scale from 1 (= I am poor at this) to 5 (= I am extremely good at this). Higher scores indicate better interpersonal competence.

In the present study, after authorization from the author of the ICQ-15, three researchers who were proficient in both Chinese and English independently translated the scale to obtain a preliminary Chinese version. Two certified translators further ensured that the translated scale was accurate and conformed to Chinese language habits. Next, it was back-translated by an English-speaking professional, and then a native English speaker evaluated its semantic equivalence. Finally, through convenience sampling, thirteen students (five college students, two high school students, three middle school students, and three elementary school students) were recruited to assess the scale’s readability. A developmental psychologist skilled in English was asked to review the final Chinese version of the scale. We provide the final Chinese version of the ICQ-15 for adolescents in the Appendix A (Table A1).

#### 2.3.2. Five-Item World Health Organization Well-Being Index (WHO-5)

The WHO-5 was developed by the World Health Organization to test subjective well-being in adults [32] and was further validated as a measure of well-being assessment among adolescents [33]. The scale consists of five items, scored on a six-point Likert scale from 0 (= at no time) to 5 (= all of the time), with the total score being the sum of the five items’ scores. Higher scores indicate higher levels of psychological well-being. The Cronbach’s alpha coefficient for the scale in this study was 0.92.

#### 2.3.3. Ten-Item Connor-Davidson Resilience Scale (CD-RISC-10)

This study used the 10-item Connor-Davidson Resilience Scale (CD-RISC-10) to assess the level of psychological resilience [34]. The CD-RISC-10 was validated in Chinese population [35]. It is a 5-point Likert scale ranging from 0 (= not true at all) to 4 (= true nearly all the time), with higher scores representing higher levels of psychological resilience. The Cronbach’s alpha coefficient for this scale in this study was 0.93.

#### 2.3.4. Patient Health Questionnaire-9 (PHQ-9)

The depressive symptoms were assessed by the Patient Health Questionnaire-9 (PHQ-9) in the preceding two weeks [36]. The PHQ-9 was validated in a Chinese adolescent population, demonstrating good psychometric properties [37]. The answers were scored on a 4-point scale from 0 (= not at all) to 3 (= nearly every day), with higher scores representing higher levels of depression. The Cronbach’s alpha coefficient for the scale in this study was 0.90.

### 2.4. Statistics and Analyses

We first analyzed the data for item–total correlation. Second, a normality test was performed on the scores of the 15 items to examine whether the responses presented a normal distribution. There are sufficient research findings to support a five-factor correlated structure of the scale. Previous research has shown that confirmatory factor analysis (CFA) can be conducted on theoretically structured or mature scales without performing an exploratory factor analysis (EFA) [38]. The EFA can be applied if the CFA results are not acceptable. The full version of the ICQ was validated in Chinese adolescents [25,39], and the scores presented good structural validity as the original theoretical model proposed by Buhrmester et al. [24]. Thus, in this study, the data were analyzed by CFA to test their structural validity. For an acceptable model fit, the following goodness of fit measures were used: Comparative Fit Index (CFI) ≥ 0.90, Tucker–Lewis Index (TLI) ≥ 0.90, and root-mean-square error for approximation (RMSEA) ≤ 0.10. Fourth, as reviewed above, interpersonal competence plays an influential role in determining general well-being, psychological resilience, and depression. In this context, Pearson’s correlation analyses were performed between the ICQ-15 and these criteria variables to assess the ICQ-15 concurrent validity. Fourth, measurement invariance is a prerequisite of a scale for comparison between different groups [40]. Hence, two multi-group analyses of the ICQ-15 were then conducted to examine the measurement invariance of this scale across genders and stages of adolescent development. Specifically, participants aged 10–13 years were grouped into early adolescence, participants aged 14–17 years were grouped into middle adolescence, and participants between the age of 18 years and early adulthood were grouped into late adolescence [31,41]. We examined configural invariance first, followed by metric invariance, scalar invariance, and strict variance. A configuration invariance test determines if the same factor structure applies to different groups. Metric invariance indicates whether the factor loadings are the same across groups. A scalar invariance indicates the presence of equal item intercepts across groups. Invariance of residual variance across groups is defined as strict invariance. The cutoff criteria for measurement invariance were 0.02 for the change of CFI and 0.03 for the change for RMSEA, as suggested by Rutkowski and Svetina for large-size samples [42]. The CFA and multi-group analyses were performed using a robust maximum likelihood (MLR) estimator. Finally, the internal consistency reliability of the ICQ-15 was examined. This study used SPSS 23.0 and Mplus 8.3 for data analyses.

## 3. Results

### 3.1. Item Analyses

The item–total correlation was analyzed by Pearson correlation. The results showed that the item–total correlation coefficients ranged from 0.57 to 0.77 (*ps* < 0.001). The normality test showed the kurtosis and skewness of the scores of the ICQ-15 items were from −1.01 to 0.08 (Table 1), i.e., within the cutoff score of ±1.50 [43]. The results suggested the items’ scores followed an approximately normal distribution.

### 3.2. Structural Validity

The results of the CFA showed that the model fitted well, with the standard loading coefficients of the items on the factors ranging from 0.64 to 0.91 (see Table 2). The fit indices were χ^2^ = 524.49, *df* = 80, CFI = 0.95, TLI = 0.93, and RMSEA = 0.06, and all met the psychometric criteria.

### 3.3. Concurrent Validity

The results of concurrent validity analyses showed that the scores of the total and five subscales of the ICQ-15 showed a moderate to strong correlation with the scores of the WHO-5 and the CD-RISC-10 (*r* = 0.51, *r* = 0.53; *ps* < 0.001) and a negative correlation with the scores of the PHQ-9 with moderate strength (*r* = −0.32; *p* < 0.001). Detailed data are presented in Table 3.

### 3.4. Multi-Group Analyses

Two multi-group analyses were conducted to test for measurement invariance among males and females, as well as in different age groups. After adding restrictions, the change of model fit indices (CFI and RMSEA) were less than the cutoff criteria of 0.020 between the male and the female groups (See Table 4) [42]. The results indicated that the ICQ-15 scale had good measurement invariance when comparing the male and the female groups. Details are shown in Table 4.

As a result of the multi-group analysis, the change in the fit indexes of CFI, TLI, and RMSEA between the participants in early, middle, and late adolescence was less than the cutoff criteria of 0.020 [42], indicating that the ICQ-15 scale has acceptable measurement invariance across these development periods. Details are shown in Table 5.

### 3.5. Internal Consistency Reliability Analyses

The Cronbach’s α coefficient for the scores of the ICQ-15 was 0.93. The Cronbach’s α coefficients for the scores of the five subscales (i.e., initiation of a relationship, negative assertion, disclosure, emotional support, and conflict management) were 0.81, 0.85, 0.80, 0.84, and 0.73, respectively. The McDonald’s ω coefficient for the scores of the ICQ-15 was 0.93, and that for the scores of the five subscales was 0.81, 0.87, 0.80, 0.84, and 0.73 in the same sequential as above, respectively.

## 4. Discussion

The purpose of this study was to examine the applicability of the ICQ-15 among Chinese adolescents and to enrich the measurement tools for improving interpersonal competence targeted at Chinese adolescents. The findings indicated the ICQ-15 has satisfactory item–total correlation and discrimination. The CFA showed an acceptable model fit index. The multi-group analyses showed that there was measurement invariance across males and females as well as across different stages of adolescence. The results supported our research hypothesis. Consistent with previous research [25,26,27,28,29,30], this study supports the interpersonal relationship competency scale consisting of five dimensions: initiation of a relationship, negative assertion, disclosure, emotional support, and conflict management.

The findings of the concurrent validity analysis showed that adolescents’ interpersonal competence was positively correlated with subjective well-being and psychological resilience at a medium to high intensity, i.e., the stronger the interpersonal competence, the better the subjective well-being and psychological resilience, which is similar to the findings of previous studies [13,44]. This may be because adolescents are at a crucial transitional period in their development, where the influence of peer relationships becomes more prominent than in childhood. Adolescents with good interpersonal competence are likely to have higher levels of social support and benign emotional support. Therefore, they are more likely to feel connected with others, which will lead to a higher self-determination and more satisfaction in their lives [44]. In addition, when faced with distress situations, adolescents with high interpersonal competence can better regulate their emotions with the support of their families and peers, which will contribute to constructive stress coping strategies and better adaption. Thus, individuals with strong interpersonal competence may have a greater sense of well-being and psychological resilience in life. Interpersonal competence was also significantly and negatively associated with mental health problems, which is similar to the findings of previous studies [9,10]. The authors speculated that adolescents with poor interpersonal competence could face more negative interpersonal stress. Chronic negative stress can result in the impairment of adolescents’ self-concept, endocrine system (e.g., cortisol dynamics), and nervous systems, ultimately affecting their psychological and even biological health [45].

The Cronbach’s alpha coefficient for the ICQ-15 was 0.93, and the Cronbach’s alpha coefficients of the five dimensions (initiation of a relationship, negative assertion, disclosure, emotional support, and conflict management) were 0.81, 0.85, 0.80, 0.84, and 0.73, respectively. Thus, the results indicated that the ICQ-15 has good internal consistency reliability, similar to the findings of a previous study [17]. Further, in the present study, after adding restrictions to the structural model of the ICQ-15, the subsequent change values of the fit indices all fell below 0.01, indicating that the Chinese version of the ICQ-15 scale has good measurement invariance across the male and female student groups and between the 10–24 age group.

There are some strengths of this study. First, the present study sample broadly covered all stages of adolescence, with good age representation. In addition, the Chinese version of the ICQ-15 validated in this study provides a concise and practical measurement for assessing adolescents’ interpersonal competence. This is conducive to future interpersonal competence evaluation, behavioral experiments, or interventions. Third, this study found that adolescents with better interpersonal competence had higher subjective well-being and psychological resilience and fewer depressive symptoms, suggesting the importance of enhancing adolescents’ interpersonal competence in education and clinical work. Cultivating the interpersonal competence of adolescents can promote their healthy development and enhance their adaptability. Overall, the ICQ-15 has good reliability and validity in the Chinese adolescent population and can be used as a scientific tool to measure the interpersonal skills of Chinese adolescents.

Several limitations to this study need to be noted. First, the subject group was mainly from Shenzhen City rather than a nation-wide sample. Future studies could increase the sample size including participants from other regions to minimize the geographical homogeneity of the participants. In addition, all assessments used in the present study were self-reported. Future studies could enrich the validity criteria by incorporating more assessment dimensions (other-rated scales, e.g., peer- or teacher-rated scales). Third, due to the constraints of the study sampling, the retest data could not be collected in this study. Thus, the test–retest reliability could not be obtained, which could be complemented in future studies.

## 5. Conclusions

This study assessed the reliability and validity of the ICQ-15 among Chinese adolescents. Item analyses, confirmatory factor analysis, concurrent validity analyses, multi-group analyses, and internal consistency analyses were performed on the scores of the ICQ-15 among 1705 Chinese adolescents. The results suggested good psychometric features of the Chinese version of the ICQ-15 which can be used as a tool for assessing interpersonal competence among Chinese adolescents.

## Figures and Tables

**Table 1:** Kurtosis and skewness of the scores of the ICQ-15 items (*n* = 1705).

Item	1	2	3	4	5	6	7	8	9	10	11	12	13	14	15
Skewness	−0.16	−0.42	−0.20	−0.47	−0.20	−0.38	−0.17	−0.43	−0.22	−0.13	−0.69	0.08	−0.03	−0.52	−0.47
Kurtosis	−0.55	−0.52	−0.98	−0.30	−0.98	−0.47	−0.95	−0.66	−1.01	−0.79	−0.01	−1.00	−0.96	−0.22	−0.66

**Table 2:** Standardized factor loadings for the five-factor model of the ICQ-15 (*n* = 1705).

Initiation of Relationship		Negative Assertion		Disclosure		Emotional Support		Conflict Management	
Item	Loadings	Item	Loadings	Item	Loadings	Item	Loadings	Item	Loadings
2	0.77	10	0.64	3	0.70	4	0.80	1	0.65
7	0.79	12	0.91	5	0.79	11	0.79	6	0.82
9	0.73	13	0.91	8	0.79	14	0.81	15	0.60

**Table 3:** Correlation coefficients between the ICQ-15 and other related measures and inter-factor correlations between ICQ-15 factors (*n* = 1705).

	WHO-5	CD-RISC-10	PHQ-9	F1	F2	F3	F4	F5
F1	0.50 *	0.47 *	−0.33 *					
F2	0.40 *	0.39 *	−0.24 *	0.59 *				
F3	0.44 *	0.41 *	−0.30 *	0.74 *	0.52 *			
F4	0.39 *	0.47 *	−0.24 *	0.68 *	0.56 *	0.64 *		
F5	0.36 *	0.46 *	−0.22 *	0.59 *	0.48 *	0.55 *	0.71 *	
ICQ-15	0.51 *	0.53 *	−0.32 *	0.88 *	0.77 *	0.84 *	0.86 *	0.79 *

Note. *: Significant at the 0.001 level (two-tailed); WHO-5: Well-Being Index Scale; CD-RISC-10: 10-item Connor–Davidson Resilience Scale; PHQ-9: Patient Health Questionnaire-9; ICQ-15: Brief form of the Interpersonal Competence Scale. F1: initiation of a relationship; F2: negative assertion; F3: disclosure; F4: emotional support; F5: conflict management.

**Table 4:** Results of the multi-group analysis between males and females (*n* = 1705).

	χ²	*df*	CFI	TLI	RMSEA	△CFI	△RMSEA
Configural MI	651.354	160	0.944	0.927	0.060	-	-
Weak MI	693.361	175	0.941	0.929	0.059	0.003	−0.001
Strong MI	764.039	190	0.935	0.928	0.060	0.006	0.001
Strict MI	786.243	205	0.934	0.932	0.058	0.001	−0.002

Note. CFI: Comparative Fit Index. TLI: Tucker–Lewis Index. RMSEA: Root-Mean-Square Error for Approximation. MI: Measurement Invariance.

**Table 5:** Results of the multi-group analysis between different stages of adolescent development.

	χ²	*df*	CFI	TLI	RMSEA	△CFI	△RMSEA
Configural MI	768.704	240	0.944	0.926	0.062	-	-
Weak MI	863.323	270	0.937	0.926	0.062	−0.007	0.000
Strong MI	1027.317	300	0.923	0.919	0.065	−0.014	−0.007
Strict MI	1176.197	330	0.910	0.914	0.067	−0.013	−0.005

Note. CFI: Comparative Fit Index. TLI: Tucker–Lewis Index. RMSEA: Root-Mean-Square Error for Approximation. MI: Measurement Invariance.

## Data Availability

The data of this study are available in the open science framework (https://osf.io/27whc/ (accessed on 10 October 2022)).

## Appendix A

**Table A1:** Chinese Version of the Brief form of Interpersonal Competence Questionnaire for Adolescents.

	比较困难 1	勉强可以 2	基本可以 3	擅长 4	非常擅长 5
1. 当和好朋友的分歧快要发展成激烈争吵时，能够承认你可能是错的。					
2. 寻找一些事情，邀请你认为有趣和有吸引力的新朋友一起去做。					
3. 信任你的新朋友或者交往对象，让他/她看到你更柔软、敏感的一面。					
4. 帮助一个好朋友认识到他/她正在经历的问题的关键。					
5. 让一个新朋友了解“真实的”你。					
6. 在争吵中能够站在朋友的角度思考，真正理解他/她的观点。					
7. 向你可能想认识或交往的人介绍你自己。					
8. 卸下防御的“铠甲”，信任你的好朋友。					
9. 主动联系新朋友/熟人，约个时间聚一起做一些事。					
10. 当你的好朋友违背承诺时，能够与他/她直面矛盾。					
11. 当好朋友情绪低落时，能够说一些话或者做一些事情来支持他/她。					
12. 当朋友做了伤害你感情的事，能告诉他/她。					
13. 当同伴/熟人做了让你生气的事，能告诉他/她。					
14. 当你的好朋友需要帮助和支持时，能够以容易被对方接受的方式给到建议。					
15. 为了避免破坏关系的冲突，不对好朋友大发脾气(即使你更占理)。					
